# Abstracts from the 6th Respiratory Effectiveness Group Summit, 18–20 March, 2021

**DOI:** 10.1186/s12931-021-01915-5

**Published:** 2022-02-18

## PP01 Medication use and COPD control status based on clinical and CAT criteria

### Anthony D'Urzo^**1**^, Marc Miraviltes^2^, Pawel Sliwinski^3^, Chin Kook Rhee^4^, Rhichard Costello^5^, Jessica Tan^6^, Victoria Carter^7^, Therese Sophie Lapperre^8^, Bernardino Alcazar^9^, Caroline Gouder^10^, Cristina Esqinas^11^, Juan Luis garcia Rivero^12^, Juan José Soler Cataluña^13^, Anu Kemppinen^14^, Augustine Tee^15^, Miguel Roman Rodríguez^16^, David Price^17^

#### ^1^University of Toronto, Toronto, Canada, ^2^Pneumology Department, University Hospital Vall d’Hebron, Barcelona, Spain, ^3^Institute of Tuberculosis and Lung Diseases, Warsaw, Poland, ^4^University of Korea, Seoul, Republic of Korea, ^5^Royal College of Surgeons in Ireland, Dublin, Ireland, ^6^Optimum Patient Care, Cambridge, United Kingdom, ^7^Singapore General Hospital, Singapore, ^8^Duke-National University of Singapore, Singapore, ^9^Bispebjerg Hospital, Copenhagen, Denmark, ^10^Hospital de Alta Resolución de Loja, Spain, ^11^Mater Dei Hospital, Malta, ^12^Hospital Comarcal de Laredo, Cantabria, Spain, ^13^Respiratory and Critical Care Medicine, Changi General Hospital, Singapore, ^14^Primary Health-care Center Son Pisà. IB-Salut, Palma, Spain, ^15^Pneumology Department, Hospital Arnau de Vilanova, Valencia, Spain, ^16^Centre of Academic Primary Care, University of Aberdeen, United Kingdom, ^17^Observational and Pragmatic Research Institute, Singapore

##### **Correspondence:** Anthony D'Urzo

*Respiratory Research* 2021, **22(1)**: PP01

**Rationale:** In a recent report (1) control status by clinical criteria (CC) was noted to be a better predictor of exacerbations compared to the COPD Assessment Test (CAT) criteria and that control was more likely to be achieved using clinical compared to CAT criteria. In the present report we describe medication use and COPD control based on clinical and CAT criteria.

**Methods:** This is a post-hoc cross-sectional analysis of data of the REG control prospective international study. A total of 307 patients were analysed (mean age 68.6 years and mean FEV1(%)= 52.5%).

**Results:** See attached results tables.

Medication use and COPD control based on CAT.

Based on Clinical and CAT Criteria, forced expiratory volume in one second (FEV1) and forced vital capacity (FVC) in controlled patients was greater in individuals receiving LAMA alone compared to LABA/LAMA/ICS, P<0.002. While no differences were noted in the uncontrolled CC group for FEV1 and FVC, FEV1 in the CAT group was higher in the LAMA vs LABA/LAMA/ICS group, p<0.02. Values for mMRC in both the CAT and CC groups were significantly higher in the LABA/LAMA/ICS vs LAMA with the exception of those uncontrolled in the CC group where there was no difference.

**Conclusions:** Our findings show that there appears to be differences in COPD control based on CC and medication group warrants further study in larger primary care populations.

## Reference

1. Soler-Cataluña JJ, Marzo M, Catalán P, Miralles C, Alcazar B, Miravitlles M. Validation of clinical control in COPD as a new tool for optimizing treatment. Int J Chron Obstruct Pulmon Dis 2018; 13: 3719-3731.

**Funding**

The study was funded by an unrestricted grant from Novartis AG.

**Results:** Medication use and COPD control based on CAT Criteria*

**Table:** 

Medication	**Controlled** **(n=116)**	**Uncontrolled** **(n=190)**	**P-value**
SABA/SAMA alone or in combination	4 (3.4%)	6 (3.2%)	0.890
LABA alone	11 (9.5%)	21 (11.1%)	0.663
LAMA alone	20 (17.2%)	20 (10.5%)	0.091
ICS alone	0 (0%)	1 (0.5%)	0.434
LABA/LAMA	36 (31%)	43 (22.6)	0.103
LABA/ICS	15 (12.9%)	25 (13.2%)	0.285
LAMA/ICS	0 (0%)	6 (3.2%)	0.053
LABA/LAMA/ICS	29 (25%)	66 (34.7%)	0.074

Medication use and COPD control based on Clinical Criteria*

**Table:** 

Medication	**Controlled** **(n=197)**	**Uncontrolled** **(n=106)**	**P-value**
SABA/SAMA alone or in combination	6 (3%)	4 (3.8%)	0.735
LABA alone	24 (12.2%)	8 (7.5%)	0.211
LAMA alone	32 (16.2%)	7 (6.6%)	**0.017**
ICS alone	1 (0.5%)	0 (0%)	0.462
LABA/LAMA	55 (27.9%)	23 (21.7%)	0.238
LABA/ICS	23 (11.7%)	17 (16%)	0.285
LAMA/ICS	4 (2%)	2 (2%)	0.932
LABA/LAMA/ICS	49 (24.9%)	45 (42.5%)	**0.002**

**Disclosures**:

Dr. D’Urzo has received research, consulting and lecturing fees from GlaxoSmithkline, Sepracor, Schering Plough, Altana, Methapharma, AstraZeneca, ONO pharma, Merck Canada, Forest Laboratories, Novartis Canada/USA, Boehringer Ingelheim (Canada) Ltd, Pfizer Canada, SkyePharma, and KOS Pharmaceuticals and Almirall, Sanofigenzyme.

## PP02 Improving the Assessment of Adults with Chronic Cough in Primary Care

### Alan Kaplan

#### ^1^Family Physician Airways Group of Canada, Canada

##### **Correspondence:** Alan Kaplan

*Respiratory Research* 2021, **22(1)**: PP02

**Research question:** What are the essential and achievable elements required to support methodical assessment and referral of chronic cough in adults seen in primary care?

**Background:** Chronic cough (>8 weeks) is a common reason for patient visits to primary care physicians (PCPs). Careful assessment of chronic cough is critical, because it can mask more serious conditions and has a significant impact on patient well-being and quality of life. Multiple guidelines encompass the assessment of chronic cough by specialists,1,2 but there is less information available for the primary care setting. We have developed a simplified algorithm for the assessment of chronic cough in adult patients in Canadian primary care, modeled on the American College of Chest Physicians (ACCP) guidelines1. The aim of our proposed study is to further refine and validate this algorithm.

**Possible methodology:** We propose to refine the algorithm through presentations at conferences and to other groups of primary care physicians and specialists. Feedback from these settings will be used to modify the algorithm, with the goal of emphasizing assessment elements that can be achieved by primary care physicians prior to (and even during the process of) referral to specialty care. We anticipate the development of related versions of this algorithm, tailored to reflect local or national practice patterns and testing/specialist access. Validation of the algorithm could be achieved by examining the proportion of chronic cough patients within primary care who were successfully evaluated or referred before, versus after implementation of the algorithm by primary care physicians who choose to use the algorithm in routine clinical care.

**Questions to discuss:** The proposed study will help us identify assessment elements required for a successful diagnosis or referral of chronic cough in primary care patients. The use of the assessment algorithm has the potential to improve the care of patients with chronic cough, by ensuring appropriate work-up/assessment of a patient is not delayed whilst referral to secondary care is being sought. Supporting a patient through what can be a long and complex disease management process, has the potential to improve patient quality of life and associated journey.

**Declaration of interest:** Dr. Kaplan is on advisory board or speakers bureau for Astra Zeneca, Behring, Boehringer Ingelheim, Covis, Griffols, GSK, Merck Frosst, Pfizer, Purdue, Novartis, NovoNordisk, Sanofi, Teva and Trudel

**References**

1. Irwin RS et al. Chest 2018;153:196-209.
2. Morice AH et al. Eur Respir J 2020;55:pii: 1901136.

## PP03 Estimating the Economic Value of Pipeline Chronic Cough Therapies

### R. Brett McQueen^1^, Kunal Saxena^2^, Jonathan Schelfhout^2^, Melanie Whittington^1^, Jonathan Campbell^1^

#### ^1^University of Colorado, Aurora, United States, ^2^Merck & Co., Kenilworth, United States

##### **Correspondence:** R. Brett McQueen

*Respiratory Research* 2021, **22(1)**: PP03

**Introduction:** Globally, more than $10 billion is spent annually on the treatment of chronic cough. Multiple pipeline therapies for the treatment of refractory chronic cough (RCC) are forthcoming and will need economic value evidence for coverage and reimbursement recommendations. Our objective was to build an economic modeling framework to identify a range of economic value scenarios using conservative and optimistic clinical benefits derived from early phase evidence on RCC pipeline therapies versus usual care (e.g., anti-tussive medications, corticosteroids, antibiotics, etc.).

**Methods:** The proposed modeling framework for RCC includes health states “on treatment” and “off treatment” for both treatment arms, defined by treatment on active therapy and active therapy discontinuation back to usual care (Figure). The model approach links changes in cough frequency as defined by early phase clinical trials (i.e., 24-hr cough frequency) with direct and indirect costs, and health-related quality of life (HRQoL) utility scores. RCC intervention costs were not available at the time of this analysis. In lieu of comprehensive trial evidence at the time of this abstract deadline, inputs were derived from early phase trials, expert opinion, and asthma proxies (controlled and partially controlled vs. uncontrolled) for changes in utility and direct and indirect cost offsets. Outcomes from the hypothetical model emphasize cost-offsets from the U.S. societal perspective and incremental quality-adjusted life years (QALYs) over a lifetime. Costs and outcomes were discounted at 3% per year.

**Results:** Simulated patient cohorts were similar to early phase trial populations with a mean age of 60, a mean (SD) 24-hr cough frequency of 27.5 (19.6), and discontinuation from active therapy of 20.6% within the first 3 months. On average, 9.8 years on active therapy was modeled over a lifetime. Assuming similar HRQoL utility and cost relationships to changes in asthma control, reducing 24-hr cough frequency by 45% (conservative clinical benefit), may result in an additional 0.26 QALYs with cost offsets of $16,000 over a lifetime compared to usual care alone. Whereas reducing 24-hr cough frequency by 60% (optimistic clinical benefit) may result in an additional 0.62 QALYs with cost offsets of $22,000 over a lifetime compared to usual care.

**Conclusions:** Future evidence generation should link cough frequency with improvements in day-to-day symptom management, work productivity, and HRQoL utility. Comprehensive economic assessments will also include the costs of RCC therapies alongside measures such as incremental QALYs and cost offsets.
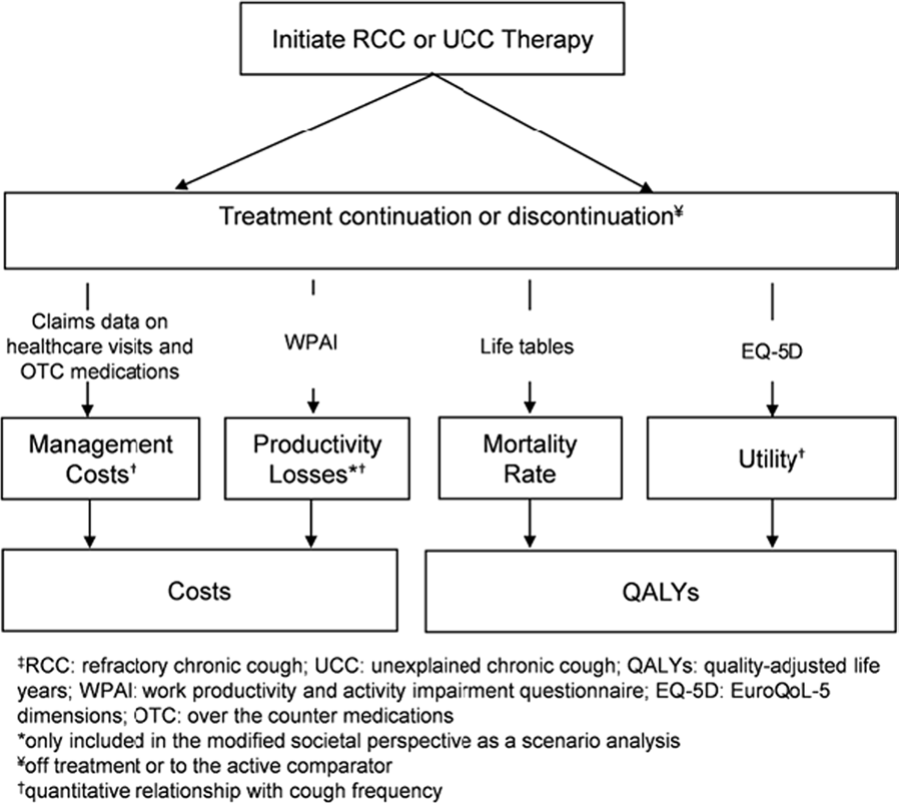


**Disclosures:**

RBM, MDW, and JDC received consulting fees from Merck & Co. to support this work. KS and JS are employees of Merck & Co.

## PP04 Eliciting Patient-Informed Value Elements for Economic Evaluation of COPD Treatment

### Julia Slejko^1^, Yoon Duk Hong^1^, Laura Bozzi^1^, Susan dosReis^1^

#### ^1^University of Maryland School of Pharmacy, Baltimore, United States

##### **Correspondence:** Julia Slejko

*Respiratory Research* 2021, **22(1)**: PP04

**Introduction.** There is increasing interest in patient-centered economic evaluations and methods to incorporate the patient’s perspective. Our previous work elicited 44 patient-informed value elements (i.e. factors related to healthcare that are important to patients) by directly engaging patients across a range of conditions. The objective of this study was to develop and test a discrete choice experiment (DCE) to quantify value elements specific to patients with chronic obstructive pulmonary disease (COPD).

**Methods:** Twenty-three study participants diagnosed with COPD completed four guided activities and a demographic questionnaire, administered through in-person, telephone or video interviews. Participants were asked to select specific elements that were important to them among three categories: treatment-, outcome- and care process-related factors. For the elements that emerged as most important, individual video interviews were conducted with seven participants to establish the attributes and wording for inclusion in a DCE instrument. A pre-test of the DCE instrument was conducted with ten participants.

**Results:** Interviews with 23 COPD patients resulted in eight value elements that emerged as most important, including four treatment-related, one care process-related, and three outcome-related attributes. Feedback from seven participants resulted in the addition of one care process-related attribute and consolidation and/or substitution of outcome- and treatment-related attributes. This resulted in the selection of six attributes for the instrument: two care process-related (Access to Care, Explanation of Benefits & Risks), three treatment-related (Side Effects, New Therapeutic Option, Willingness to Pay), and one outcome-related (Physical Endurance). A balanced orthogonal design with 100% D-efficiency was used to construct a DCE with nine experimentally derived choice tasks, each with three profiles displaying six attributes per profile. Two hold-out choice tasks were added as a reliability test.

**Conclusion:** A patient-informed economic evaluation begins with understanding elements of value from the patient perspective. Patient inclusion in the qualitative development of stated preference instruments authentically quantifies patient preferences. Resulting preference weights reflect the relative importance of patient-informed value elements. The next phase of this research will apply preference weights in a patient-informed economic evaluation.

## PP05 Long-acting anti-muscarinic agents (LAMA) frequency of use and clinical features of patients with severe asthma in real-life setting: data from the Severe Asthma Network in Italy (SANI) registry

### Enrico Heffler^1,2^, Francesca Puggioni^1,2^, Giorgio Walter Canonica^1,2^, Francesco Blasi^3^, Pierluigi Paggiaro^4^, Luisa Brussino^5^, Marco Caminati^6^, Gianenrico Senna^6^

#### ^1^Humanitas University, Pieve Emanuele , Italy, ^2^Humanitas Clinical and Research Center IRCCS , Rozzano, Italy, ^3^Fondazione IRCCS Ca' Granda Ospedale Maggiore Policlinico Internal Medicine Department, Respiratory Unit and Cystic Fibrosis Adult Center, and Department of Pathophysiology and Transplantation, University of Milan, Milano, Italy, ^4^Department of Surgery, Medicine, Molecular Biology and Critical Care, University of Pisa, Pisa, Italy, ^5^University of Torino, Torino, Italy, ^6^Asthma Center and Allergy Unit, Verona University Hospital, Verona, Italy

##### **Correspondence:** Enrico Heffler

*Respiratory Research* 2021, **22(1)**: PP05

**Introduction:** Patients with uncontrolled asthma despite high doses of inhaled corticosteroid plus another controller are defined as severe asthmatics. Tiotropium bromide Respimat is the only long acting muscarinic agonists (LAMA) approved for severe asthma.

**Aims:** To explore the frequency of severe asthmatics treated with LAMAs and characterize their clinical features in a real-life, registry-based setting.

**Methods:** Baseline data from the Severe Asthma Network in Italy (SANI) registry have been analyzed to study the use of LAMA and possible clinical features associated to it in severe asthmatics.

**Results:** Among a total of 698 enrolled patients, 35.9% were treated with LAMAs (23.3% Tiotropium bromide Respimat, 4.5% Tiotropium bromide Handihaler, 4.5% Aclidinium, 3.4% Glycopyrronium bromide 0,3% Umeclidinium bromide). Patients taking LAMAs had higher age of

asthma onset and were more frequently former smokers. They had higher annual exacerbation rate, worst asthma control, worst disease-related quality of life and poorer lung function. Bronchiectasis were more frequently found in LAMA users (25.9% vs 13.1%).

**Conclusions:** Tiotropium bromide is still underused in severe asthma in a real-life setting, while a relevant proportion of patients are treated with other LAMAs not approved for severe asthma treatment. Patients taking LAMAs have features of the most severe asthmatics.

**Disclosures** ***:***

Personal fee and/or grants:

- Giorgio Walter Canonica: Menarini, Alk-Abellò, Anallergo, Boehringer Ingelheim, Chiesi, Circassia, Genentech, Guidotti Malesci, GSK, Meda, Merck, Merck Sharp & Dome, Novartis, Recordati-InnuvaPharma, Roche, Sanofi, Stallergenes, UCB Pharma, Uriach Pharma, Teva, AstraZeneca, ThermoFischer, Valeas, Vibor Pharma
- Francesco Blasi: AstraZenca, Bayer, Chiesi, Guidotti, GSK, Grifols, Insmed, Menarini, Novartis, Pfizer, Zambon
- Pierluigi Paggiaro: AstraZeneca, Chiesi, Novartis, Alk-Abellò, GSK, Mundipharma, Guidotti, Menarini, Sanofi
- Gianenrico Senna: AstraZeneca, GSK, Menarini, Novartis, Sanofi, Mylan
- Enrico Heffler: AstraZeneca, Sanofi, Novartis, GSK, Teva, Valeas, Circassia, Nestlè Purina

## PP06 Chronic rhinosinusitis with nasal polyps impacts in severe asthma patients: evidences from the Severe Asthma Network Italy (SANI) Registry

### **Enrico Heffler**^1,2^, Luca Malvezzi^2^, Francesco Blasi^3^, Pierluigi Paggiaro^4^, Marco Mantero^3^, Gianenrico Senna^5^, Giorgio Walter Canonica^1,2^

#### ^1^Humanitas University, Pieve Emanuele, Italy, ^2^Humanitas Clinical and Research Center IRCCS, Rozzano, Italy, ^3^Fondazione IRCCS Ca' Granda Ospedale Maggiore Policlinico Internal Medicine Department, Respiratory Unit and Cystic Fibrosis Adult Center, and Department of Pathophysiology and Transplantation, University of Milan, Milano, Italy, ^4^Department of Surgery, Medicine, Molecular Biology and Critical Care, University of Pisa, Pisa, Italy, ^5^Asthma Center and Allergy Unit, Verona University Hospital, Verona, Italy

##### **Correspondence:Enrico Heffler**

*Respiratory Research* 2021, **22(1)**: PP06

**Introduction:** The clinical and laboratory features of patients enrolled in the Severe Asthma Network in Italy (SANI) registry, a web-based observatory collecting demographic, clinical, functional and inflammatory data of patients with severe asthma were evaluated, with a special emphasis to chronic rhinosinusitis with nasal polyposis (CRSwNP).

**Methods:** For each eligible patients the following information has been collected: demographic data, clinical features, asthma control in the previous month according to the GINA (Global INitiative for Asthma) Guidelines and standardized questionnaires, concomitant regular and on demand treatments and inflammatory markers.

**Results**. 695 patients with severe asthma enrolled in 66 SANI centers were analyzed. The prevalence of chronic rhinosinusitis with nasal polyposis was 40.6%. Atopic dermatitis and bronchiectasis was significantly more frequent in patients with CRSwNP than in subjects without nasal polyposis; similarly, FeNO values are significantly higher in subject with CRSwNP respect patients without nasal polyposis. Finally, patients with CRSwNP had a significantly higher number of asthma exacerbations per year, although on more days on oral corticosteroids (OCS ) and a higher number OCS long term users.

**Conclusion:** OCS sparing is needed in patients with severe asthma, mainly in subjects with CRSwNP, adopting adequate strategies such as a better adherence to the treatment with inhaled therapy accordingly to the GINA recommendations, the use of biologic agents and a multidisciplinary approach of the patient.

**Disclosures**:

Personal fees and/or grants:

- Giorgio Walter Canonica: Menarini, Alk-Abellò, Anallergo, Boehringer Ingelheim, Chiesi, Circassia, Genentech, Guidotti Malesci, GSK, Meda, Merck, Merck Sharp & Dome, Novartis, Recordati-InnuvaPharma, Roche, Sanofi, Stallergenes, UCB Pharma, Uriach Pharma, Teva, AstraZeneca, ThermoFischer, Valeas, Vibor Pharma
- Luca Malvezzi: Sanofi-Genzyme, Novartis
- Francesco Blasi: AstraZenca, Bayer, Chiesi, Guidotti, GSK, Grifols, Insmed, Menarini, Novartis, Pfizer, Zambon
- Pierluigi Paggiaro: AstraZeneca, Chiesi, Novartis, Alk-Abellò, GSK, Mundipharma, Guidotti, Menarini, Sanofi
- Marco Mantero: Zambon, GSK, Pfizer, AstraZeneca, MedicAir, Boehringer Ingelheim, Menarini, Vivisol
- Gianenrico Senna: AstraZeneca, GSK, Menarini, Novartis, Sanofi, Mylan
- Enrico Heffler: AstraZeneca, Sanofi, Novartis, GSK, Teva, Valeas, Circassia, Nestlè Purina

## PP07 Community management of allergic rhinitis: A real life community pharmacy study targeting the barriers to Allergic Rhinitis management

### Rachel House^1,2^, Biljana Cvetkovski^1,2^, Vicky Kritikos^1,2^, Kwok Yan^2,3^, **Sinthia Bosnic-Anticevich**^1,2,4^

#### ^1^Woolcock Institute of Medical Research, Glebe, Australia, ^2^University of Sydney, Camperdown, Australia, ^3^Royal Prince Alfred Hospital, Camperdown, Australia, ^4^Sydney Local Health District, Campsie, Australia

##### **Correspondence:** Sinthia Bosnic-Anticevich

*Respiratory Research* 2021, **22(1)**: PP07

**Introduction.** Allergic Rhinitis (AR) currently affects 40% of the world’s population posing a significant burden on individuals (QOL) and society. It has been established that 75% of patients with AR self-select their medication in Australian community pharmacy: 15% select optimally. This study tested the feasibility and impact of the Allergic Rhinitis Clinical Management Pathway [AR-CMaP], (ie a pharmacy AR management approach, based on an evidence-based clinical pathway and individualised for each pharmacy setting) on the AR medication selection of people with AR.

**Methods:** A mixed-methods, repeated measures study design was implemented. Baseline data collection using a researcher-administered questionnaire, enabling the evaluation of the appropriateness of the process and outcome of the patient medication selection.

Pharmacists participated in the AR-CMaP training, which was supported by a modification of the pharmacy to address the particular needs of pharmacists (pharmacy workflow etc based on pre-identified pharmacist needs) and the patients in the pharmacy. Two weeks following training and pharmacy modification, the researcher-administered questionnaire (described above) was once again implemented. Pharmacists were interviewed to gain feedback on the implementation of the pathway in their pharmacy.

**Results:** Six pharmacies enrolled in the study; 241 and 240 eligible pharmacy customers participated at baseline and follow up respectively. The majority of AR patients experienced moderate-severe symptoms. The most common product purchased was an oral antihistamine. There were no significant changes in the pharmacist- patients interaction and medication selection process post-implementation of AR-CMaP. Forty-four percent of the AR patients reporting not seeing a need for pharmacist follow-up, 26% reported it to be a doctor’s responsibility and 20% were satisfied with their self-management. Pharmacists reported that barriers to implementing AR management guidelines included not wanting to contradict a doctor’s recommendation and AR patient’s reluctance to change their treatment.

**Conclusion:** People with AR have pre-determined approaches to the management of their AR, neither seeking or wanting pharmacist involvement. Future research and strategies need to use a novel technique to address the self-management practices of patients who still continue to select sub-optimal mediation to manage their AR.

**Disclosures:**

Dr. Tan and Mrs Cvetkovski has nothing to disclose.

Dr. Kritikos reports personal fees from AstraZeneca, personal fees from GlaxoSmithKline, personal fees from Pfizer, outside the submitted work.

Dr. Yan reports personal fees from AstraZeneca, personal fees from Boehringer Ingelheim, personal fees from GlaxoSmithKline, personal fees from Meda, personal fees from Mundipharma , personal fees from Pfizer, outside the submitted work.

Prof. Bosnic-Anticevich reports personal fees from Boehringer Ingelheim, personal fees from Boehringer Ingelheim, grants from MEDA, personal fees from TEVA, personal fees from TEVA, personal fees from AstraZeneca, personal fees from GSK, outside the submitted work.

## PP08 Workflow Mapping of Nebulized COPD Therapy in In-hospital and Long-term Care (LTC) Settings in the US: a Precursor to an Observational Time and Motion (T&M) Study

### Erwin De Cock^1^, Grace Leung^2^, **Grant Maclaine**^3^, Hemal Shah^4^, Brooks Kuhn^5^

#### ^1^Syneos Health, Real World & Late Phase, Barcelona, Spain, ^2^Theravance Biopharma (TBPH) US Inc., San Francisco, United States, ^3^TBPH Ireland Limited, Dublin, Ireland, ^4^Value Matters, LLC, United States, ^5^UC Davis School of Medicine, Sacramento, United States

##### **Correspondence:** Grant Maclaine

*Respiratory Research* 2021, **22(1)**: PP08

**Introduction:** The economic burden of COPD is substantial with medical costs projected to rise to $49 billion by 2020. Standard of care includes SABA, SAMA, or SABA+SAMA. Approximately 9% of US COPD patients use nebulizers for ongoing maintenance therapy. However, there is a lack of understanding of healthcare professional (HCP) time dedicated to nebulized COPD therapy administration. Workflow mapping was performed as a precursor to an observational T&M study.

**Methods:** A survey was designed to understand (1) center characteristics and pharmacologic COPD therapy, (2) SABA (albuterol (ALB)) and SABA+SAMA (ipratropium bromide plus albuterol sulfate (IPR/ALB)) nebulization workflow, and (3) estimated time per nebulization. Two HCPs from in-hospital and two from LTC settings completed the survey and were subsequently interviewed.

**Results:** HCPs across both settings reported that the majority of COPD patients are prescribed IPR/ALB for short-term relief. No differences in workflow were reported between IPR/ALB and ALB. There appeared to be consensus on consecutive activities; minor deviations included the need or not for pre- and post-nebulization assessment, and the logistics around storing/discarding/cleaning materials (Table). The process is performed by respiratory therapists in the in-hospital setting and by (licensed vocational) nurses in LTC. Estimated time per nebulization was 13 and 27 minutes in in-hospital setting, and 21 and 37 minutes in LTC.

Nebulization process

1. Collect nebulized drug (in-hospital automated dispensing cabinet vs. drug cart in LTC)
2. Collect materials (sometimes together with step 1)
3. Pre-nebulization assessment (may include patient education)
4. Add medication to reservoir and connect to nebulizer (may include patient education)
5. Start nebulization (may include pre-nebulization assessment)
6. Monitoring patient during nebulization
7. End nebulization (may include post-nebulization assessment; may be combined with step 8)
8. Store nebulizer/discard materials/clean nebulizer
9. Post-nebulization assessment
10. Record-keeping

**Conclusion:** Nebulization workflow is highly standardized and expected to be similar between in-hospital and LTC settings and also between nebulized drugs. Opinion-based time estimates suggest that HCPs dedicate substantial time to nebulization. This research confirmed the feasibility and suitability of T&M as a method to accurately quantify time dedicated by HCPs to perform nebulized COPD therapy in both settings. Data from this ongoing T&M study will be used to estimate potential efficiencies that could result from nebulized COPD therapies with less frequent dosing regimens.

**Table:** 

**Nebulization process**
1.Collect nebulized drug (in-hospital automated dispensing cabinet vs. drug cart in LTC)
1.Collect materials (sometimes together with step 1)
2.Pre-nebulization assessment (may include patient education)
3.Add medication to reservoir and connect to nebulizer (may include patient education)
4.Start nebulization (may include pre-nebulization assessment)
5.Monitoring patient during nebulization
6.End nebulization (may include post-nebulization assessment; may be combined with step 8)
7.Store nebulizer/discard materials/clean nebulizer
8.Post-nebulization assessment
9.Record-keeping

**Disclosures**:

*Erwin De Cock is an employee of Syneos commissioned by TBPH to conduct this project. Grant Maclaine is an employee of TBPH. Grace Leung is a consultant for TBPH. Hemal Shah is a consultant for Mylan Specialty L.P. Brooks Kuhn is a paid consultant for Syneos.*

## PP09 Opinions of GPs regarding their role in preventive medicine and eHealth support

### **Esther Metting**^1^, Nina Scheenhart^1^

#### ^1^University Of Groningen and University Medical Center Groningen, Groningen, The Netherlands

##### **Correspondence:Esther Metting**

*Respiratory Research* 2021, **22(1)**: PP09

**Background:** In 2019 the Dutch government started with reimbursing a “Combined Lifestyle Intervention-program (CLI)” for high risk patients with poor lifestyle. This is relevant for the COPD population because 88% suffers from ≥1 comorbidities which are mostly lifestyle related and these increase the risk of exacerbations1.

**Aim.** To evaluate opinions of GPs regarding their role in preventive medicine. Moreover, we evaluated the current status of the CLI in the north of the Netherlands and the possible role of eHealth in preventive medicine.

**Methods:** We performed semi structured interviews in 15 GPs (mean age=54±10 years, 87% male). The topics were: 1) opinions regarding the role of primary care, 2) views on the CLI and 3) opinions regarding eHealth. We triangulated the findings in an questionnaire with 94 GPs (mean age 52±8 years, 59% male).

Results: There was no consensus about the role of GPs in primary prevention, however secondary prevention was considered to be a task for primary care. Some GPs were demotivated “the patients’ attitude makes me give up.” Only few GPs used the CLI, because the CLI is not available in all areas. eHealth is hardly used by GPs, but is considered to be possibly relevant in a limited group of patients.

**Conclusion:** GPs are divided about their role. COPD patients with poor lifestyle might benefit from the CLI but this is not available in certain regions. Better National organisation of preventive programs and possibly innovative eHealth tools might enhance lifestyle support in primary care.

1.1. Westerik JA, Metting EI, van Boven JF, Tiersma W, Kocks JW, Schermer TR. Associations between chronic comorbidity and exacerbation risk in primary care patients with COPD. Respir Res. 2017;18(1):31. Published 2017 Feb 6. https://doi.org/10.1186/s12931-017-0512-2

## PP10 Smart spacer supported medication adherence management in patients with asthma: study protocol for the randomized controlled OUTER SPACE trial

### **Boudewijn Dierick**^1,2,3^, Susanne van de Hei^2,4,5^, Paul Hagedoorn^6^, Sandra Been-Buck^7^, Titia Klemmeier^7^, Tanja Zijp^3^, Daan Touw^3^, Huib Kerstjens^8^, Janwillem Kocks^2,4^, Job van Boven^2,3^

#### ^1^Department of General Practice & Elderly Care Medicine, University Medical Center Groningen, University of Groningen, Groningen, The Netherlands, ^2^Groningen Research Institute for Asthma and COPD (GRIAC, Groningen, The Netherlands, ^3^Department of Clinical Pharmacy & Pharmacology, University Medical Center Groningen, University of Groningen, Groningen, The Netherlands, ^4^General Practitioners Research Institute, Groningen, The Netherlands, ^5^Department of Health Sciences, , University Medical Center Groningen, University of Groningen, Groningen, The Netherlands, ^6^Department of Pharmaceutical Technology & Biopharmacy, University of Groningen, Groningen, The Netherlands, ^7^Department of Pulmonary Diseases Martini , Groningen, The Netherlands, ^8^Department of Pulmonary Diseases, University Medical Center Groningen, University of Groningen, Groningen, The Netherlands

##### **Correspondence:** Boudewijn Dierick

*Respiratory Research* 2021, **22(1)**: PP10

**Introduction:** Adherence to inhalation medicines is still a topic of major concern.This study aims to assess overall feasibility of undertaking a definitive randomized controlled trial (RCT) of a smart spacer device in adults with asthma treated in primary care with inhaled corticosteroids/long-acting beta agonists (+/-long-acting muscarinic antagonists) using a pressurized metered dose inhaler (pMDI). In particular, we aim to: 1) determine an estimated recruitment time for a RCT, 2) assess patient and healthcare provider satisfaction with the smart spacer, 3) explore the distribution of medication adherence patterns (persistence and inhaler technique) and clinical outcomes and 4) obtain data to calculate the sample size for a definitive RCT.

**Methods:** The CE-marked smart spacer used in this study is based upon the Aerochamber Plus® with Flow Vu®. The smart spacer monitors both adherence and inhaler technique and can be used with multiple pMDI devices.

Randomized controlled feasibility trial of 2 months. Patients will be recruited from four general practices in the Netherlands. Patients (n=40) will use the smart spacer for 1 month (t=-1). At t=0, they will be randomized into two groups. The intervention group will receive tailored feedback and education on the basis of data from the smart spacer; the control group will receive usual care. After 1 month (t=1), the study ends and outcomes are assessed.

**Results:** At t=-1, t=0 and t=1, ACQ, WPAI, TAI and FeNO are measured. At t=0 and t=1, lung function will be tested. At t=1, device usability is evaluated by the SUS questionnaire as well as structured interviews with patients and healthcare providers. Finally, a scalp hair sample will be taken to compare electronically collected data with long-term inhaled drug exposure.

**Conclusion:** This study will provide insight in how healthcare providers can objectively monitor and manage patients’ adherence to inhalation medicines using a smart spacer. Furthermore, we will obtain data regarding optimal outcomes for a full RCT including medication adherence, inhaler technique and clinical outcomes. This RCT will provide evidence on the potential of personalized, smart spacer-data-informed inhaler education
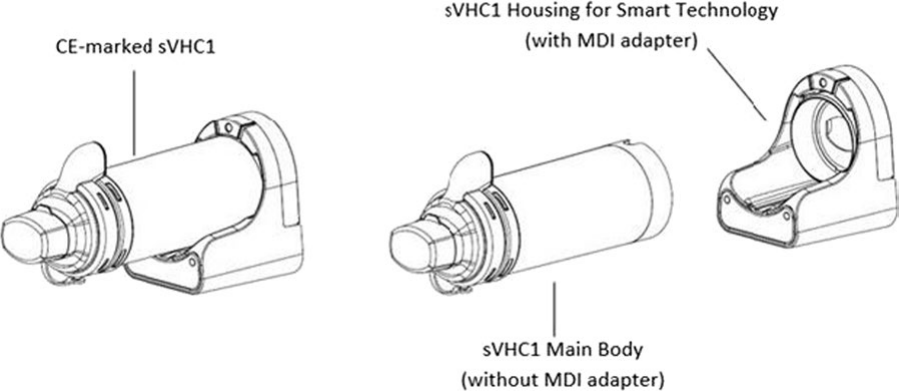


## PP11 Are COPD prescription patterns aligned with guidelines? A Canadian population-based study

### **Taraneh Bahremand**^1^, Mahyar Etminan^2^, Nardin Roshan-Moniri^1^, Mary De Vera^1^, Hamid Tavakoli^1^, Mohsen Sadatsafavi^1^

#### ^1^Collaboration for Outcomes Research and Evaluation, Faculty of Pharmaceutical Sciences, The University of British Columbia, Vancouver, Canada, ^2^Department of Ophthalmology, Faculty of Medicine, The University of British Columbia, Vancouver, Canada

##### **Correspondence:** Taraneh Bahremand

*Respiratory Research* 2021, **22(1)**: PP11

**Background:** In contemporary guidelines for the management of Chronic Obstructive Pulmonary Disease (COPD), the history of acute exacerbations plays an important role in the choice of long-term inhaled therapies. This study aimed at evaluating population-level trends of filled inhaled prescriptions over the time course of COPD and their relation to the history of exacerbations.

**Method:** We used administrative health databases in British Columbia, Canada (1997–2015) to create a retrospective incident cohort of individuals with diagnosed COPD. We quantified long-acting inhaled medication within each year of follow-up and documented its trend over the time course of COPD. Using generalized linear models, we investigated the association between the frequent exacerbator status (≥2 moderate or ≥1 severe exacerbation(s) in the previous 12 months) and filling a prescription after a physician visit.

**Results:** 132,004 COPD patients were included (mean age 68.6, 49.2% female). The most common medication class during the first year of diagnosis was inhaled corticosteroids (ICS, used by 49.9%), followed by long-acting beta-agonists (LABA, 31.8%). Long-acting muscarinic agents (LAMA) were the least commonly prescribed (10.4%). ICS remained the most common prescription throughout follow-up, being used by approximately 50% of patients during each year. 39.0% of patients received combination inhaled therapies in their first year of diagnosis, with ICS+LABA being the most common (30.7%). The association between exacerbation history was the most pronounced for triple therapy with an odds ratio (OR) of 2.68 for general practitioners (GPs) and 2.02 for specialists (internist and respirologists) (p<0.001 for both). Such associations were generally stronger among GPs compared with specialists, with the exception of monotherapy with LAMA or ICS as shown in the figure.

**Conclusion:** We documented low utilization of monotherapies (specifically LAMA) and high utilization of combination therapies (particularly ICS containing). Specialists were less likely to consider exacerbation history in the choice of inhaled therapies compared with GPs.

Figure. Forest plot of Odds Ratio (OR) and 95% confidence interval between frequent-exacerbator status and filled prescriptions for each medication type, separately for GP and specialist.

Abbreviations: ICS, inhaled corticosteroids; LABA, long-acting beta-2 adrenoceptor agonists; LAMA, long-acting muscarinic agents; GP, General practitioner.
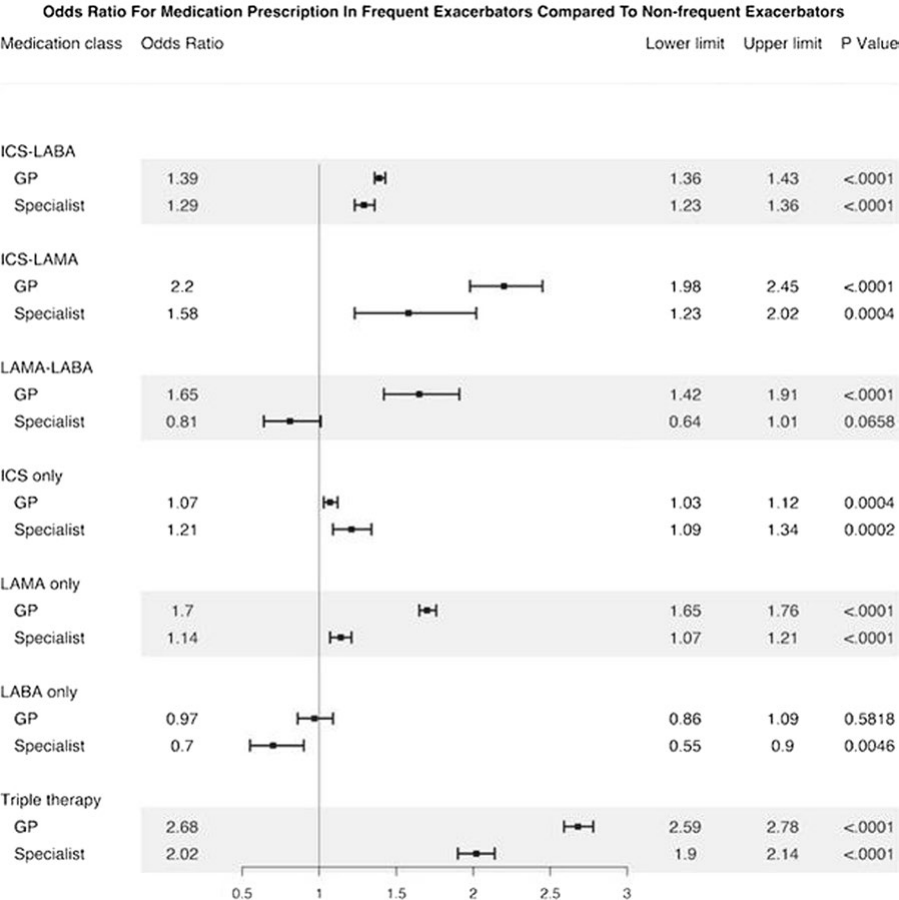


**Disclosures:**

*Dr. Mohsen Sadatsafavi is the correspondent author and has received speaker fees and honoraria from Boehringer Ingelheim, GlaxoSmithKline, and AstraZeneca. He has received research funds directly into his research accounts within The University of British Columbia from Boehringer Ingelheim and AstraZeneca.*

## PP12 Development of a tool to measure the clinical response to biologic therapy in uncontrolled severe asthma: the FEOS score.

### Luis Pérez De Llano^**1**^, Ignacio Dávila^2^, Eva Martínez Moragon^3^, Javier Dominguez Ortega^4^, Carlos Almonacid^5^, Carlos Colás^6^, Juan Luis García-Rivero^7^, Borja G Cosio^8^

#### ^1^Pneumology Service. Lucus Augusti University Hospital, Lugo, Spain, ^2^Department of Allergy, University Hospital of Salamanca., Salamanca, Spain, ^3^Pneumology Service. Hospital Universitario Dr Peset. Valencia, Spain, ^4^La Paz Hospital Institute for Health Research (IdiPAZ), Department of Allergy Madrid, Spain. CIBER of Respiratory Diseases CIBERES, Spain. Madrid, Spain, ^5^Pneumology Service. Hospital Universitario de Toledo, Toledo, Spain, ^6^Hospital Clínico-Instituto de Investigación Sanitaria de Aragón. Zaragoza, Spain, ^7^Department of Respiratory Medicine. Hospital de Laredo. Laredo, Spain, ^8^Department of Respiratory Medicine. Hospital Universitario Son Espases-IdISBa-Ciberes. Palma de Mallorca, Spain

##### **Correspondence:** Luis Pérez De Llano

*Respiratory Research* 2021, **22(1)**: PP12

**Background:** There is a lack of tools to holistically quantify the response to monoclonal antibodies (mAbs) in severe uncontrolled asthma (SUA) patients. The aim of this study was to develop a valid score to assist specialists in this clinical context.

**Methods:** The score was developed in 4 subsequent phases: (1) elaboration of the theoretical model of the construct intended to be measured (response to mAbs); (2) definition and selection of items and measurement instruments by Delphi survey; (3) weight assignment of the selected items by multicriteria decision analysis (MCDA) using the Potentially All Pairwise RanKings of all possible Alternatives (PAPRIKA) methodology via the 1000Minds software; and (4) face validity assessment of the obtained score.

**Results:** Four core items, with different levels of response for each of them, were selected: “severe exacerbations”, “oral corticosteroid use”, “symptoms” (evaluated by Asthma Control Test: ACT) and “bronchial obstruction” (assessed by FEV1 % theoretical). “Severe exacerbations” and “oral corticosteroid maintenance dose” were weighted most heavily (38% each), followed by “symptoms” (13%) and “FEV1” (11%). Higher scores in the weighted system indicate better response and the range of responses runs from 0 (worsening) to 100 (best possible response). Face validity was high (intraclass correlation coefficient: 0.86).

**Conclusions:** The FEOS score (FEV1, Exacerbations, Oral corticosteroids, Symptoms) allows clinicians to quantify response in SUA patients who are being treated with mAbs.
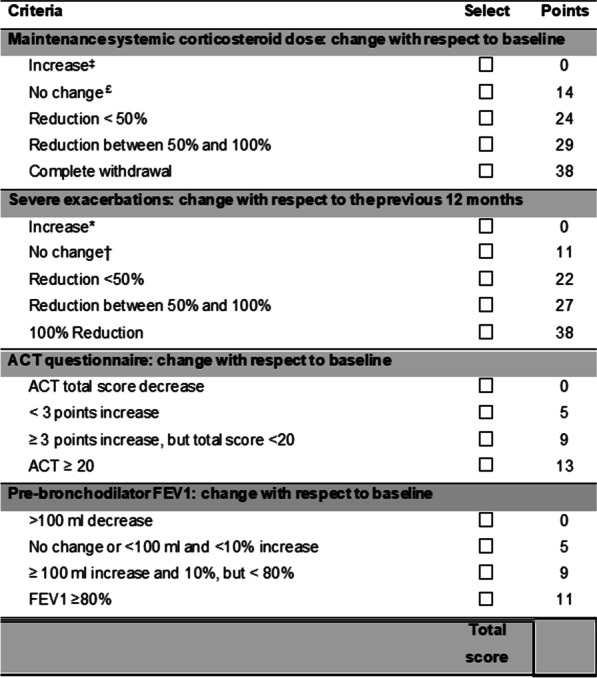


**Disclosures** ***:***

*LPLL reports grants, personal fees and non-financial support from AstraZeneca, personal fees and non-financial support from GSK, grants and personal fees from TEVA, personal fees and non-financial support from Novartis, personal fees and non-financial support from Chiesi, personal fees and non-financial support from Boehringer, personal fees from Sanofi, personal fees from Menarini, personal fees and non-financial support from Mundipharma, grants and personal fees from Esteve, personal fees from ROVI, personal fees from BIAL, personal fees from MSD, personal fees from TECHDOW PHARMA, non-financial support from FAES, outside the submitted work. In the last five years, ID has received speaker’s honoraria from AstraZeneca, Novartis, TEVA, Sanofi/Regeneron, Chiesi, and GSK, and honoraria for attending advisory panels with Sanofi/Regeneron, AstraZeneca, GSK, Chiesi, and Novartis. EMM received honoraria for speaking at sponsored meetings from AstraZeneca, Boehringer-Ingelheim, Chiesi, GlaxoSmithKline, Novartis, TEVA and ALK; and as a consultant for AstraZeneca, Boehringuer-Ingelheim, TEVA and GlaxoSmithKline. JDO reports personal fees for consultant and lectures from ALK, AstraZeneca, Letipharm, Mundipharma, Chiesi, Novartis, GlaxoSmithKline, MSD, Sanofi and TEVA, outside the submitted work. CA has participated in speaking activities, advisory committees and consultancies sponsored by: AstraZeneca, Boehringer-Ingelheim, Chiesi, Menarini, GSK, ALK Mundipharma, Novartis, Pfizer, TEVA, SEPAR and NEUMOMADRID. CAS declares not receiving ever, directly or indirectly, funding from the tobacco industry or its affiliates. CC reports having served as a consultant to Mylan and AstraZeneca; having been paid lecture fees by AstraZeneca, GSK, Mylan, MSD, grants from Roxall, from Novartis, AstraZeneca, and Sanofi. JLGR received speaking or advisory fees, or economic aid, to attend congresses, or participation in clinical studies on behalf of (alphabetical order): ALK, Astra-Zeneca, Boehringer-Ingelheim, Chiesi, Laboratorios Ferrer, GlaxoSmithKline, Menarini, Novartis, Rovi, and TEVA, and consulting fees from AstraZeneca, Boehringer-Ingelheim, GlaxoSmithKline, Grifols, Menarini, and Novartis. CBG reports grants, personal fees and non-financial support from GSK, grants, personal fees and non-financial support from Chiesi, grants, personal fees and non-financial support from Astrazeneca, grants from Menarini and Boehringer-Ingheilm, non-financial support from Novartis, personal fees and non-financial support from Sanofi, outside the submitted work.*

## PP13 Biologic Utilization Patterns: Data from the International Severe Asthma Registry (ISAR)

### Andrew Menzies-Gow^**1**^, Prof Eileen Wang^2,3^, Mari-Anne Rowlands^4^, Marianna Alacqua^5^, Mona Al-Ahmad^6^, Lakmini Bulathsinhala^4^, Victoria Carter^4^, Isha Chaudhry^4^, Borja Cosio^7^, Neva Eleangovan^4^, J. Mark FitzGerald^8^, Liam Heaney^9^, Mark Hew^10,11^, Naeimeh Hosseini^4^, David Jackson^12,13^, Maria Kallieri^14^, Désirée Larenas Linnemann^15^, Stelios Loukides^14^, Njira Lugogo^16^, Ruth Murray^4^, Andriana Papaioannou^14^, Luis Perez-de-Llano^17^, Celeste Porsbjerg^18^, Linda M. Rasmussen^19^, Johannes Schmid^20^, Trung Tran^5^, Charlotte Ulrik^21^, John Upham^22^, David Price^4,23,24^

#### ^1^UK Severe Asthma Network and National Registry, Royal Brompton & Harefield NHS Foundation Trust, London, UK, ^2^Division of Allergy & Clinical Immunology, Department of Medicine, National Jewish Health, USA, ^3^Division of Allergy & Clinical Immunology, Department of Internal Medicine, University of Colorado Hospital, USA, ^4^Optimum Patient Care, UK, ^5^AstraZeneca, Gaithersburg, USA, ^6^Al-Rashed Allergy Center, Ministry of Health, Microbiology Department, Faculty of Medicine, Kuwait University, Kuwait, ^7^Son Espases University Hospital-IdISBa-Ciberes, Spain, ^8^Centre for Lung Health, Canada, ^9^UK Severe Asthma Network and National Registry, Queen’s University Belfast, Northern Ireland, ^10^Allergy, Asthma & Clinical Immunology Service, Alfred Health, Australia, ^11^Public Health and Preventive Medicine, Monash University, Australia, ^12^UK Severe Asthma Network and National Registry, Guy's and St Thomas' NHS Trust, UK, ^13^School of Immunology & Microbial Sciences, King's College London, UK, ^14^2nd Respiratory Medicine Department, National and Kapodistrian University of Athens Medical School, Attikon University Hospital, Greece, ^15^Directora Centro de Excelencia en Asma y Alergia, Hospital Médica Sur, Ciudad de México, Mexico, ^16^Department of Medicine, Division of Pulmonary and Critical Care Medicine, University of Michigan, Ann Arbor, USA, ^17^Department of Respiratory Medicine, Hospital Universitario Lucus Augusti, Spain, ^18^Respiratory Research Unit, Bispebjerg University Hospital, Denmark, ^19^Department of Respiratory Medicine, Bispebjerg and Frederiksberg Hospital, Denmark, ^20^University Hospital of Aarhus, Denmark, ^21^Department of Respiratory Medicine, Hvidovre Hospital, Denmark, ^22^Diamantina Institute & PA-Southside Clinical Unit, The University of Queensland, Australia, ^23^Observational and Pragmatic Research Institute, Singapore, ^24^Centre of Academic Primary Care, Division of Applied Health Sciences, University of Aberdeen, UK

##### **Correspondence:** Andrew Menzies-Gow

*Respiratory Research* 2021, **22(1)**: PP13

**Introduction:** Use of biologics in severe asthma has grown dramatically in the last decade. However, little is known about the patterns of biologic use in real-life. Our aim was to describe frequency and patterns of biologic use in an international severe asthma cohort.

**Methods:** The International Severe Asthma Registry (ISAR; http://isaregistries.org) launched in 2017 includes patients aged ≥18 years on Global INitiative for Asthma (GINA) Step 5 or GINA Step 4 treatment with uncontrolled symptoms. Severe asthma patients recruited between January 2015 to August 2019 from Bulgaria, Canada, Greece, Italy, Japan, Kuwait, South Korea, Spain, and the United States (US) were included in the analysis (n=6,477). All countries had licences for ≥2 biologics. The following biologic utilization patterns were captured: 1) persistence on biologic for ≥6 months, 2) stopping (no record of biologic use for ≥3 months after the end of the last prescription), or 3) single switch/multiple switches (received a biologic, followed by a switch to another biologic). Both retrospective and prospective medication records were considered.

**Results:** Of the 6,477 patients with severe asthma, 1,727 were treated with biologics during 2017 to 2019. Of these patients, 73% (n=1,255) persisted with their biologic, 16% (n=280) stopped, and 9% (n=151) switched once or twice to a second or third biologic. Biologic persistence was most prevalent in Italy and least prevalent in Japan. More patients in the US (27%) stopped their biologic compared to other countries. South Korea had the most patients (33%) who switched biologics, although absolute numbers were low. Of those who switched once to a second biologic (n=122), 84% (n=103) continued on the second biologic. Only 11% (n=16) of 151 patients who switched once switched again to a third biologic, and of those 75% (n=12) persisted on the third biologic.

**Conclusion:** At the time of this data cut, three-quarters of patients with a biologic prescription were maintained on the first biologic therapy, with only a small percentage stopping or switching to another biologic. The majority of those who switched persisted with their second biologic, with only a very small percentage progressing to a third biologic. Patterns of use may be driven by multiple factors such as 1) biologic availability, 2) biologic prescription requirements, 3) country-specific health system issues, 4) patient preference and expectations, and 5) national stopping guidelines. These factors should be considered in future work analysing usage patterns.
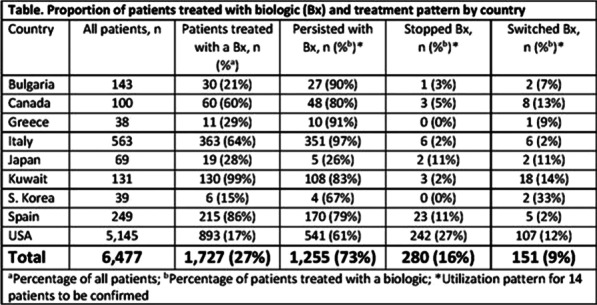


**Disclosures:**

Andrew N. Menzies-Gow has attended advisory boards for AstraZeneca, GlaxoSmithKline, Novartis, Sanofi and Teva, and has received speaker fees from AstraZeneca, Novartis, Roche, Teva and Sanofi. He has participated in research with AstraZeneca for which his institution has been remunerated and has attended international conferences with Teva. He has had consultancy agreements with AstraZeneca, Sanofi, and Vectura.

Eileen Wang has received honoraria from AstraZeneca and Clinical Care Options. She has been an investigator on clinical trials sponsored by AstraZeneca, GlaxoSmithKline, Genentech, Novartis, Teva, and National Institute of Allergy and Infectious Diseases (NIAID) for which her institution has received funding.

Mari-Anne Rowlands, Lakmini Bulathsinhala, Victoria Carter, Isha Chaudhry, Neva Eleangovan, Naeimeh Hosseini are employees of Optimum Patient Care, a co-funder of the International Severe Asthma Registry.

Marianna Alacqua and Trung N. Tran are employees of AstraZeneca, a co-funder of the International Severe Asthma Registry.

Mona Al-Ahmad has received advisory board and speaker fees from AstraZeneca, Sanofi, Novartis, and GlaxoSmithKline.

Borja G. Cosio declares grants from Chiesi and GSK; personal fees for advisory board activities from Chiesi, GSK, Novartis, Sanofi and AstraZeneca; and payment for lectures/speaking engagements from Chiesi, Novartis, GSK, Menarini, and AstraZeneca, outside the submitted work.

J. Mark FitzGerald, Maria Kallieri, Linda M. Rasmussen, Johannes Schmid, and Ruth Murray declare no relevant conflicts of interest.

Liam G. Heaney declares he has received grant funding, participated in advisory boards and given lectures at meetings supported by Amgen, AstraZeneca, Boehringer Ingelheim, Circassia, Evelo Biosceinces, Hoffmann la Roche, GlaxoSmithKline, Novartis, Theravance and Teva; he has taken part in asthma clinical trials sponsored by Boehringer Ingelheim, Hoffmann la Roche, and GlaxoSmithKline for which his institution received remuneration; he is the Academic Lead for the Medical Research Council Stratified Medicine UK Consortium in Severe Asthma which involves industrial partnerships with a number of pharmaceutical companies including Amgen, AstraZeneca, Boehringer Ingelheim, GlaxoSmithKline, Hoffmann la Roche, and Janssen.

Mark Hew declares grants and other advisory board fees (made to his institutional employer) from AstraZeneca, GlaxoSmithKline, Novartis, and Seqirus, for unrelated projects.

David J. Jackson has received advisory board and speaker fees from AstraZeneca, GlaxoSmithKline, Boehringer Ingelheim, Teva, Napp, Chiesi, Novartis and research grant funding from AstraZeneca.

Désirée Larenas Linnemann reports personal fees from Amstrong, AstraZeneca, Boehringer Ingelheim, Chiesi, DBV Technologies, Grunenthal, GSK, MEDA, Menarini, MSD, Novartis, Pfizer, Novartis, Sanofi, Siegfried, UCB, Alakos, Gossamer, grants from Sanofi, AstraZeneca, Novartis, UCB, GSK, TEVA, Boehringer Ingelheim, Chiesi, and Purina institute, outside the submitted work.

Stelios Loukidis received honorarium form Novartis, Astra, GSK; received grants from GSK.

Njira Lugogo consulted for AstraZeneca and GSK; on protocol committee with AstraZeneca; on advisory board with AstraZeneca, GSK, Sanofi, Novartis, Genentech and Teva.

Andriana I. Papaioannou has received fees and honoraria from Menarini, GSK, Novartis, Elpen, Boehringer Ingelheim, AstraZeneca, and Chiesi.

Luis Perez-de-Llano declares non-financial support, personal fees, and grants from Teva; non-financial support and personal fees from Boehringer Ingelheim, Esteve, GlaxoSmithKline, Mundipharma, and Novartis; personal fees and grants from AstraZeneca and Chiesi; personal fees from Sanofi; and non-financial support from Menairi outside the submitted work.

Celeste M. Porsbjerg has attended advisory boards for Astra Zeneca, Novartis, TEVA, and Sanofi-Genzyme; has given lectures at meetings supported by Astra Zeneca, Novartis, TEVA, Sanofi-Genzyme, and GlaxoSmithKline; has taken part in clinical trials sponsored by Astra Zeneca, Novartis, MSD, Sanofi-Genzyme, GlaxoSmithKline, and Novartis; and has received educational and research grants from Astra Zeneca, Novartis, TEVA, GlaxoSmithKline, ALK, and Sanofi-Genzyme.

Charlotte S. Ulrik has attended advisory boards for AstraZeneca, ALK-Abello, GSK, Boehringer-Ingelheim, Novartis, Chiesi, TEVA, Actelion and Sanofi-Genzyme; has given lectures at meetings supported by AstraZeneca, Sandoz, Mundipharma, Chiesi, Boehringer-Ingelheim, Orion Pharma, Novartis, TEVA, Sanofi-Genzyme, and GlaxoSmithKline; has taken part in clinical trials sponsored by AstraZeneca, Novartis, Chiesi, Merck, InsMed, ALK-Abello, Stallergenes, TEVA, Sanofi-Genzyme, GlaxoSmithKline, Boehringer-Ingelheim and Novartis; and has received educational and research grants from AstraZeneca, Mundipharma, Boehringer-Ingelheim, Novartis, TEVA, GlaxoSmithKline and Sanofi-Genzyme.

John W. Upham has received speaker fees and consulting fees from Novartis, Astra Zeneca, GSK, Sanofi, and Boehringer Ingelheim.

David Price has board membership with AstraZeneca, Boehringer Ingelheim, Chiesi, Mylan, Novartis, Regeneron Pharmaceuticals, Sanofi Genzyme, Thermofisher; consultancy agreements with Airway Vista Secretariat, AstraZeneca, Boehringer Ingelheim, Chiesi, EPG Communication Holdings Ltd, FIECON Ltd, Fieldwork International, GlaxoSmithKline, Mylan, Mundipharma, Novartis, OM Pharma SA, PeerVoice, Phadia AB, Spirosure Inc, Strategic North Limited, Synapse Research Management Partners S.L., Talos Health Solutions, Theravance and WebMD Global LLC; grants and unrestricted funding for investigator-initiated studies (conducted through Observational and Pragmatic Research Institute Pte Ltd) from AstraZeneca, Boehringer Ingelheim, Chiesi, Mylan, Novartis, Regeneron Pharmaceuticals, Respiratory Effectiveness Group, Sanofi Genzyme, Theravance and UK National Health Service; payment for lectures/speaking engagements from AstraZeneca, Boehringer Ingelheim, Chiesi, Cipla, GlaxoSmithKline, Kyorin, Mylan, Mundipharma, Novartis, Regeneron Pharmaceuticals and Sanofi Genzyme; payment for travel/accommodation/meeting expenses from AstraZeneca, Boehringer Ingelheim, Mundipharma, Mylan, Novartis, Thermofisher; stock/stock options from AKL Research and Development Ltd which produces phytopharmaceuticals; owns 74% of the social enterprise Optimum Patient Care Ltd (Australia and UK) and 92.61% of Observational and Pragmatic Research Institute Pte Ltd (Singapore); 5% shareholding in Timestamp which develops adherence monitoring technology; is peer reviewer for grant committees of the UK Efficacy and Mechanism Evaluation programme, and Health Technology Assessment; and was an expert witness for GlaxoSmithKline.

## PP14 Exacerbations Are Associated with Lung Function Decline in a Broad Asthma Population in England, Scotland, and Wales 1950-2019

### Seyi Soremekun^1,2^, Liam G. Heaney^3^, Derek Skinner^1,2^, Lakmini Bulathsinhala^1,2^, Victoria Carter^1,2^, Isha Chaudhry^1,2^, Naeimeh Hosseini^1,2^, **Neva Eleangovan**^1,2^, Ruth Murray^1,2^, Trung N. Tran^4^, Benjamin Emmanuel^4^, Esther Garcia Gil^5^, Andrew Menzies-Gow^6^, Matthew Peters^7^, Njira Lugogo^8^, Rupert Jones^2,9^, David Price^1,2,10^

#### ^1^Optimum Patient Care, Cambridge, United Kingdom, ^2^Observational and Pragmatic Research Institute, Singapore, ^3^UK Severe Asthma Network and National Registry, Queen’s University Belfast, Northern Ireland, ^4^AstraZeneca, Gaithersburg, USA, ^5^AstraZeneca, Spain, ^6^UK Severe Asthma Network and National Registry, Royal Brompton & Harefield NHS Foundation Trust, UK, ^7^Department of Thoracic Medicine, Concord Hospital, Australia, ^8^Department of Medicine, Division of Pulmonary and Critical Care Medicine, University of Michigan, Ann Arbor, USA, ^9^Faculty of Medicine & Dentistry, University of Plymouth, UK, ^10^Centre of Academic Primary Care, Division of Applied Health Sciences, University of Aberdeen, UK

##### **Correspondence:** Neva Eleangovan

*Respiratory Research* 2021, **22(1)**: PP14

**Introduction:** Progressive lung function decline in patients with asthma may result in poorer control and worsening quality of life. Asthma exacerbations are thought to contribute to this decline. However, evidence is mixed and limited to a few mainly small, post-hoc studies. This longitudinal study aimed to assess the association between exacerbation burden and long-term lung function decline in a broad asthma patient population.

**Methods:** This was a historical cohort study of a broad asthma patient population covering the United Kingdom in the Optimum Patient Care Research Database. Patients were followed up from the first eligible post-18th birthday peak expiratory flow rate (PEF) record (primary analysis), or record of forced expiratory flow in 1 second (sensitivity analysis) until the last record of the same type. Linear growth models that adjusted for age, sex, follow-up length, height, and time-varying smoking status were used to test the impact of mean annual exacerbation rate (AER - averaged over follow-up) on lung function trajectory both overall and stratified by age (18-24, 25-39 and 40+ years) and by mean dosage of inhaled corticosteroids (ICS), categorised into terciles (lowest, middle and highest).

**Results:** We studied 109,182 patients with follow-up between 5 and 60 years. For each additional exacerbation per year an estimated additional 0.21% predicted PEF/year was lost (95% CI 0.18, 0.25). The effect was greatest in younger adults where those with AERs of 2+ and aged 18-24 years at baseline lost an additional 1.27% predicted PEF/year (95% CI 0.73, 1.81) compared to those with AER 0. These differences in the rate of LF decline between AER groups became progressively smaller as age at baseline increased. Apart from patients in the lowest ICS dosage tercile where there was no significant impact, there was a significant acceleration in lung function decline in patients with higher AERs compared to AER 0 for those in the middle and highest ICS terciles. The results using FEV1 were consistent with the above.

**Conclusion:** To our knowledge, this is the largest, population-based assessment of asthma exacerbation burden and lung function decline and addresses key evidence gaps. We show that exacerbations are associated with faster lung function decline, which is most accelerated in patients aged under 40 years and not entirely prevented by ICS. Earlier intervention with appropriate management in younger asthma patients could be of value to prevent excessive lung function decline.

**Disclosures:**

Seyi Soremekun, Derek Skinner, Lakmini Bulathsinhala, Victoria Carter, Isha Chaudhry, Naeimeh Hosseini, and Neva Eleangovan are employees of Optimum Patient Care, a co-funder of the International Severe Asthma Registry.

Liam G. Heaney declares he has received grant funding, participated in advisory boards and given lectures at meetings supported by Amgen, AstraZeneca, Boehringer Ingelheim, Circassia, Evelo Biosceinces, Hoffmann la Roche, GlaxoSmithKline, Novartis, Theravance and Teva; he has taken part in asthma clinical trials sponsored by Boehringer Ingelheim, Hoffmann la Roche, and GlaxoSmithKline for which his institution received remuneration; he is the Academic Lead for the Medical Research Council Stratified Medicine UK Consortium in Severe Asthma which involves industrial partnerships with a number of pharmaceutical companies including Amgen, AstraZeneca, Boehringer Ingelheim, GlaxoSmithKline, Hoffmann la Roche, and Janssen.

Ruth Murray declares no relevant conflicts of interest.

Trung N. Tran, Benjamin Emmanuel, and Esther Garcia Gil are employees of AstraZeneca, a co-funder of the International Severe Asthma Registry.

Andrew N. Menzies-Gow has attended advisory boards for AstraZeneca, GlaxoSmithKline, Novartis, Sanofi and Teva, and has received speaker fees from AstraZeneca, Novartis, Roche, Teva and Sanofi. He has participated in research with AstraZeneca for which his institution has been remunerated and has attended international conferences with Teva. He has had consultancy agreements with AstraZeneca, Sanofi, and Vectura.

Matthew Peters declares personal fees and non-financial support from AstraZeneca and GlaxoSmithKline.

Njira Lugogo consulted for AstraZeneca and GSK; on protocol committee with AstraZeneca; on advisory board with AstraZeneca, GSK, Sanofi, Novartis, Genentech and Teva.

Rupert Jones reports grants, personal fees, and non-financial support from AstraZeneca and OPRI, personal fees and non-financial support from Boehringer Ingelheim, grants, personal fees, and non-financial support from GSK, grants and non-financial support from Novartis, non-financial support from Nutricia, and personal fees from Pfizer outside the submitted work.

*David Price has board membership with AstraZeneca, Boehringer Ingelheim, Chiesi, Mylan, Novartis, Regeneron Pharmaceuticals, Sanofi Genzyme, Thermofisher; consultancy agreements with Airway Vista Secretariat, AstraZeneca, Boehringer Ingelheim, Chiesi, EPG Communication Holdings Ltd, FIECON Ltd, Fieldwork International, GlaxoSmithKline, Mylan, Mundipharma, Novartis, OM Pharma SA, PeerVoice, Phadia AB, Spirosure Inc, Strategic North Limited, Synapse Research Management Partners S.L., Talos Health Solutions, Theravance and WebMD Global LLC; grants and unrestricted funding for investigator-initiated studies (conducted through Observational and Pragmatic Research Institute Pte Ltd) from AstraZeneca, Boehringer Ingelheim, Chiesi, Mylan, Novartis, Regeneron Pharmaceuticals, Respiratory Effectiveness Group, Sanofi Genzyme, Theravance and UK National Health Service; payment for lectures/speaking engagements from AstraZeneca, Boehringer Ingelheim, Chiesi, Cipla, GlaxoSmithKline, Kyorin, Mylan, Mundipharma, Novartis, Regeneron Pharmaceuticals and Sanofi Genzyme; payment for travel/accommodation/meeting expenses from AstraZeneca, Boehringer Ingelheim, Mundipharma, Mylan, Novartis, Thermofisher; stock/stock options from AKL Research and Development Ltd which produces phytopharmaceuticals; owns 74% of the social enterprise Optimum Patient Care Ltd (Australia and UK) and 92.61% of Observational and Pragmatic Research Institute Pte Ltd (Singapore); 5% shareholding in Timestamp which develops adherence monitoring technology; is peer reviewer for grant committees of the UK Efficacy and Mechanism Evaluation programme, and Health Technology Assessment; and was an expert witness for GlaxoSmithKline.*

## PP15 Research priorities in pediatric asthma: A global, multistakeholder survey by the Pediatric Asthma in Real Life (PeARL) Think Tank.

### **Alexander Mathioudakis**^1,2^, Adnan Custovic^3^, Antoine Deschildre^4^, Francine M. Ducharme^5^, Omer Kalayci^6^, Clare Murray^1,2^, Antonio Nieto Garcia^7^, Wanda Phipatanakul^8^, David Price^9,10^, Aziz Sheikh^11^, Ioana Agache^12^, Leonard Bacharier^13^, Matteo Bonini^14,15^, Jose A. Castro-Rodriguez^16^, Giuseppe De Carlo^17^, Timothy Craig^18^, Zuzana Diamant^19,20,21^, Wojciech Feleszko^22^, Despo Ierodiakonou^23^, James E. Gern^24^, Jonathan Grigg^25^, Gunilla Hedlin^26^, Elham M. Hossny^27^, Tuomas Jartti^28^, Alan Kaplan^29^, Robert F. Lemanske^24^, Peter Le Souef^30^, Mika J. Makela^31^, Paolo M. Matricardi^32^, Michael Miligkos^33^, Mario Morais-Almeida^34^, Helena Pite^34,35,36^, Paulo MC Pitrez^37^, Petr Pohunek^38^, Graham Roberts^39,40,41^, Sylvia Sanchez-Garcia^42^, Ioanna Tsiligianni^23^, Steve Turner^43^, Tonya A. Winders^44,45^, Gary Wong^46^, Paraskevi Xepapadaki^47^, Heather Zar^48^, Nikolaos G. Papadopoulos^1,47^

#### ^1^Division of Infection, Immunity and Respiratory Medicine, School of Biological Sciences, The University of Manchester, Manchester, UK, ^2^North West Lung Centre, Wythenshawe Hospital, Manchester University NHS Foundation Trust, Manchester Academic Health Science Centre, Manchester, UK, ^3^Department of Paediatrics, Imperial College London, UK, ^4^CHU Lille, Université Nord de France, Unité de Pneumologie et Allergologie Pédiatriques, Hôpital Jeanne de Flandre, Lille, France, ^5^Department of Pediatrics, University of Montreal, Montreal , Canada, ^6^Pediatric Allergy and Asthma Unit, Hacettepe University School of Medicine, Ankara, Turkey, ^7^Pediatric Allergy Unity, University Hospital La Fe, Valencia, Spain, ^8^Children's Hospital Boston, Pediatric Allergy and Immunology, Boston, USA, ^9^Centre of Academic Primary Care, Division of Applied Health Sciences, University of Aberdeen, Aberdeen, UK, ^10^Observational and Pragmatic Research Institute, Singapore, Singapore, ^11^Asthma UK Centre for Applied Research, Usher Institute of Population Health Sciences and Informatics, The University of Edinburgh, Edinburgh, UK, ^12^Faculty of Medicine, Transylvania University, Brasov, Romania, ^13^Division of Allergy, Immunology, and Pulmonary Medicine, Department of Pediatrics, Washington University, St. Louis, USA, ^14^Department of Cardiovascular and Thoracic Sciences, Catholic University of the Sacred Heart, Fondazione Policlinico Universitario “A. Gemelli”, Rome, Italy, ^15^National Heart and Lung Institute (NHLI), Imperial College London, London, UK, ^16^Department of Pediatrics, School of Medicine, Pontifical Universidad Catolica de Chile, Santiago, Chile, ^17^European Federation of Allergy and Airway Diseases Patient's Associations, Brussels, Belgium, ^18^Penn State Allergy, Asthma and Immunology, Hershey, USA, ^19^Department of Respiratory Medicine and Allergology, Institute for Clinical Science, Skane University Hospital, Lund University, Lund, Sweden, ^20^Department of Respiratory Medicine, First Faculty of Medicine, Charles University and Thomayer Hospital, Prague, Czech Republic, ^21^Department of Clinical Pharmacy & Pharmacology, University of Groningen, University Medical Center of Groningen and QPS-NL, Groningen, Netherlands, ^22^Department of Pediatric Pulmonology and Allergy, The Medical University Children's Hospital, Warszawa, Poland, ^23^Health Planning Unit, Department of Social Medicine, Faculty of Medicine, University of Crete, Crete , Greece, ^24^Department of Pediatrics and Medicine, University of Wisconsin School of Medicine and Public Health, Madison, USA, ^25^Centre for Genomics and Child Health, Blizard Institute, Queen Mary University of London, London, UK, ^26^Department of Women’s and Children’s Health, Karolinska Institutet, Stockholm, Sweden, ^27^Pediatric Allergy and Immunology Unit, Children’s Hospital, Ain Shams University, Cairo, Egypt, ^28^Department of Paediatrics, Turku University Hospital and University of Turku, Turku, Finland, ^29^Family Physician Airways Group of Canada, University of Toronto, Toronto, Canada, ^30^School of Paediatrics and Child Health, University of Western Australia, Perth, Australia, ^31^Helsinki University Skin and Allergy Hospital and University of Helsinki, Helsinki, Finland, ^32^Department of Pediatric Pulmonology, Immunology and Intensive Care Medicine, Charité - University Medicine Berlin, Berlin, Germany, ^33^Division of Pediatric Pulmonology, First Department of Pediatrics, National and Kapodistrian University of Athens School of Medicine and Aghia Sophia Children's Hospital, Athens , Greece, ^34^Allergy Center, CUF Descobertas Hospital, Lisbon, Portugal, ^35^Allergy Center, CUF Infante Santo Hospital, Lisbon, Portugal, ^36^Chronic Diseases Research Center (CEDOC), NOVA Medical School / Faculdade de Ciências Médicas, Universidade NOVA de Lisboa, Lisbon, Portugal, ^37^Laboratory of Respiratory Physiology, Infant Center, School of Medicine, Pontifícia Universidade Católica do Rio Grande do Sul (PUCRS), Porto Alegre, Brazil, ^38^Paediatric Department, Second Faculty of Medicine, Charles University and Motol University Hospital, Prague, Czech Republic, ^39^The David Hide Asthma and Allergy Research Centre, St Mary's Hospital, Newport Isle of Wright, UK, ^40^Faculty of Medicine, Clinical and Experimental Sciences and Human Development in Health Academic Units, University of Southampton, Southampton, UK, ^41^NIHR Biomedical Research Centre, University Hospital Southampton NHS Foundation Trust, Southampton, UK, ^42^Allergy Department, Hospital Infantil Universitario Niño Jesús, Madrid, Spain, ^43^Child Health, Royal Aberdeen Children’s Hospital and University of Aberdeen, Aberdeen, UK, ^44^Allergy and Asthma Network, Vienna, Virginia, USA, ^45^Global Allergy & Asthma Patient Platform, Vienna, Austria, ^46^Department of Paediatrics, Faculty of Medicine, The Chinese University of Hong Kong, Sha Tin, Hong Kong, ^47^Allergy Department, 2nd Paediatric Clinic, University of Athens, Athens, Greece, ^48^Department of Paediatrics and Child Health, Red Cross Children's Hospital and SA-Medical Research Council Unit on Child and Adolescent Health, University of Cape Town, Cape Town, South Africa

##### **Correspondence:** Alexander Mathioudakis

*Respiratory Research* 2021, **22(1)**: PP15

**Introduction:** Pediatric asthma remains a public health challenge with enormous impact worldwide. There is a need of high-quality research and clinical recommendations to improve clinical outcomes. Pediatric Asthma in Real Life (PeARL), a think tank led by international clinical researchers in pediatric asthma initiated by the Respiratory Effectiveness Group (REG) aims to address this issue by developing consensus and recommendations that will improve patient care and limit disease burden, and also by crowdsourcing international expertise on pediatric asthma.

We present the results of a global, multi-stakeholder survey aiming to identify and prioritize unmet clinical needs in pediatric asthma that could be used to guide future research and policy activities.

**Methods:** Unmet needs weer identified through an initial open-question survey that was administered to international experts in pediatric asthma. Prioritization of topics was then achieved through a second, extensive survey with global reach involving multiple stakeholders (leading experts, researchers, clinicians, patients, policy makers and the pharmaceutical industry).

**Results:** 57 unmet needs were identified by international experts and were prioritized by 412 survey responders from 5 continents and 60 countries.

**Conclusion:** There is agreement among different stakeholder groups in the majority of research and strategic priorities for pediatric asthma. Stakeholder diversity is crucial for highlighting divergent issues that future guidelines should consider. The PeARL Think Tank will attempt to address prioritized issues by producing focused evidence updates and by developing clinical and research recommendations.

**Disclosures:**

*This study was supported by the Respiratory Effectiveness Group (REG). None of the authors had any conflicts of interest directly related to this work. IA, MB, GDC JACR, WF, GC, GH, EMH, DI, TJ, OK, MJM, PMM, MM, MM-A, ANG, HP, PMCP, SS-G PLS, AS, ST, TW, GW, HZ do not have any conflicts of interest outside the submitted work either. AGM reports grants from Boehringer Ingelheim outside the submitted work. AC reports personal fees from Novartis, Regeneron / Sanofi, Thermo Fisher Scientific, Boehringer Ingelheim and Philips, outside the submitted work. LB reports personal fees from Aerocrine, GlaxoSmithKline, Genentech/Novartis, Merck, DBV Technologies, Teva, Boehringer Ingelheim, AstraZeneca, WebMD/Medscape, Sanofi/Regeneron, Vectura and Circassia outside the submitted work. TC reports grants and personal fees CSL Behring, Dyax, Takeda, BioCryst, Pharming, personal fees from Grifols, grants and non-financial support from GSK, Regeneron, Novartis/Genetech outside the submitted work. AD reports grants and personal fees from Stallergenes Greer, personal fees from Novartis, ALK, TEVA, GSK, MEDA-MYLAN, CHIESI, AImmune, DBV technologies and Astra Zeneca, outside the submitted work. ZD reports personal fees from academic affiliations, ZD acts as Executive and Scientific Medical Director at a phase I/II pharmacological unit (QPS-NL), which performs clinical studies for pharmaceutical companies. ZD reports personal fees from Astrazeneca, ALK, Aquilon, Boehringer Ingelheim, CSL, HAL Allergy, MSD, and Sanofi-Genzyme outside the submitted work. FMD reports grants from Thorasys Inc; personal fees from Jean-Coutu Pharmaceuticals, non-financial support from Novartis Canada, and Trudell Medical, outside the submitted work. JEG reports grants from NIH/NIAID, personal fees from Regeneron, Ena Theraputics and MedImmune outside the submitted work; personal fees and stock options from Meissa Vaccines Inc outside the submitted work. JG reports personal fees from GSK, Vifor Pharmaceuticals, Novartis, BV Pharma and AstraZeneca outside the submitted work. AK reports personal fees Astra Zeneca, Behring, Boehringer Ingelheim, Covis, GSK, NovoNordisk, Novartis, Griffols, Pfizer, Sanofi, Teva and Trudel, outside the submitted work. RFL reports grants from NIH, non-financial support from GlaxoSmithKline, Boehringer-Ingelheim, Merck, TEVA, American Academy of Allergy, Asthma and Immunology, grants from Clinical and Translational Science Award (NIH), Childhood Origins of ASThma (COAST) grant, AsthmaNet, personal fees from LSU, Elsevier, UpToDate, the University of Kentucky, ThermoFischer, and Food Allergy Research and Education (FARE) Network, outside the submitted work. CM reports personal fees from Novartis, GSK, Astra Zeneca, Thermo Fisher and Boehringer Ingelheim outside the submitted work. NGP reports personal fees from Novartis, Nutricia, HAL, MENARINI/FAES FARMA, SANOFI, MYLAN/MEDA, BIOMAY AstraZeneca, GSK, MSD, ASIT BIOTECH and Boehringer Ingelheim; grants from Gerolymatos International SA and Capricare outside the submitted work. WP reports grants from NIH; grants and personal fees from Genentech/Novartis, Sanofi/Rgeneron; personal fees GSK; non-financial support from Thermo Fisher, Lincoln Diagnostics, Alk Abello, and Monaghen, outside the submitted work. PP reports grants from Astra Zeneca, Chiesi and TEVA; personal fees from Astra Zeneca, TEVA, Novartis, Mundipharma, S&D Pharma, and GlaxoSmithKline outside the submitted work. DP reports grants from AKL Research and Development Ltd, British Lung Foundation, Respiratory Effectiveness Group and the UK National Health Service; grants and personal fees from Boehringer Ingelheim, Chiesi, Circassia, Mylan, Mundipharma, Napp, Novartis, Pfizer, Regeneron Pharmaceuticals, Sanofi Genzyme, TEVA, Theravance, and Zentiva (Sanofi Generics); personal fees from Cipla, GlaxoSmithKline, Kyorin, and Merck; non-financial support from Efficacy and Mechanism Evaluation programme, Health Technology Assessment, outside the submitted work; DP also reports stock/stock options from AKL Research and Development Ltd which produces phytopharmaceuticals; and owns 74% of the social enterprise Optimum Patient Care Ltd (Australia and UK) and 74% of Observational and Pragmatic Research Institute Pte Ltd (Singapore), outside the submitted work. GCR reports personal fees from ALK, Allergen Therapeutics, Meda Plus, Merck; and a patent for the use of sublingual immunotherapy to prevent the development of allergy in at-risk infants, outside the submitted work. IT reports personal fees from Novartis, GSK, Boehringer Ingelheim, and Astra Zeneca; grants from GSK Hellas, outside the submitted work. PX reports personal fees from Nutricia, Nestle, Friesland, Uriach, Novartis Pharma AG, and GlaxoSmithkline outside the submitted work.*

## PP16 How do young adults manage their hay fever?

### **Georgina Jones**^1,2^, Biljana Cvetkovski^1,2^, Rachel House^1,2^, Sinthia Bosnic-Anticevich^1,2,3^

#### ^1^Woolcock Institute of Medical Research, Glebe, Australia, ^2^University of Sydney, Sydney, Australia, ^3^Sydney Local Health District, Sydney, Australia

##### **Correspondence:** Georgina Jones

*Respiratory Research* 2021, **22(1)**: PP16

**Introduction:** Allergic rhinitis (AR) affects 24% of young adults (≤26 years old1-3) in Australia, making it the most common long-term chronic condition for this age group4. When suboptimally managed, AR imposes a significant burden on people’s quality of life (QOL), particularly their sleep quality and daytime productivity5,6. Furthermore, 86% people with asthma also experience AR. AR has a direct impact on asthma control and, if poorly managed, it can increase the risk of the asthma exacerbations4. The nature of AR and the way it is managed has been well researched in both adult and paediatric populations. However, there is a gap in our understanding of the way AR is managed in young adults. Given the unique biopsychosocial developmental challenges faced by young adults, it is important that we investigate the management of AR in this population. This study aims to investigate the AR status of young adults. It also aims to investigate the way young adults manage their AR and the different sources of influence on their AR management.

**Methods:** This study was carried out online using cross-sectional observational study design. This survey included 20 items and investigated 3 domains; i) AR status, ii) AR medication management and iii) influences on AR management. The data were described descriptively, and logistic regression was used to determine the factors associated with optimal AR management.

**Results:** 145 participants were recruited in this study; 94% reported AR impacting on at least one domain of QOL was burdened with general burden and study/work of most concerned and 32% have coexisting asthma. Only 11% of the participants were managing their AR with optimal treatment for the reported AR symptoms and their severity. General practitioners, pharmacists and parents had the strongest influence on participants’ AR management.

**Conclusion:** This study indicates that the majority of the young adults with AR are experiencing high burden on their QOL and are not managing their AR with appropriate treatment. As young adults transition to adult care, they require developmentally appropriate health care support to equip them with the health literacy skills needed to appropriately manage their AR.

**References**:

1. Chua et al (2013). Pediatrics 131: 892-901.
2. Davey et al (2013). MC Family Practice 14: 202.
3. Knibb et al. (2020). Allergy 75: 1880-1897.
4. AIHW (2019). Allergic rhinitis ('hay fever') Australian Institute of Health and Welfare: Canberra.
5. Meltzer et al. (2009). J Allergy Clin Immunol 124: S43-70.
6. Walker et al (2007). J Allergy Clin Immunol 120: 381-387.

**Disclosures:**

Miss Jones, Dr. Cvetkovski and Dr. House declare no conflict of interest. Prof. Bosnic-Anticevich is a member of the Teva Pharmaceuticals Devices International Key Experts Panel, has received research support from Research in Real Life, has received lecture fees and payment for developing educational presentations from Teva, GSK, AstraZeneca and Mundipharma; and has received Honoria from AstraZeneca, Boehringer Ingelheim, GlaxoSmithKline, for her contribution to advisory boards/key international expert forum.

